# Cerebrospinal fluid: a target of some fungi and an overview

**DOI:** 10.1590/0074-02760220251

**Published:** 2023-03-20

**Authors:** Danielly Corrêa-Moreira, Rodolfo Castro, Gisela Lara da Costa, Reginaldo Gonçalves Lima-Neto, Manoel Marques Evangelista Oliveira

**Affiliations:** 1 Fundação Oswaldo Cruz-Fiocruz, Instituto Oswaldo Cruz, Laboratório de Taxonomia, Bioquímica e Bioprospecção de Fungos, Rio de Janeiro, RJ, Brasil; 2 Fundação Oswaldo Cruz-Fiocruz, Escola Nacional de Saúde Pública, Rio de Janeiro, RJ, Brasil; 3 Universidade Federal do Rio de Janeiro, Instituto de Saúde Coletiva, Rio de Janeiro, RJ, Brasil; 4 Universidade Federal de Pernambuco, Recife, PE, Brasil

**Keywords:** fungal meningitis, diagnosis, cerebrospinal fluid

## Abstract

Meningitis is a potentially life-threatening infection characterised by the inflammation of the leptomeningeal membranes. The estimated annual prevalence of 8.7 million cases globally and the disease is caused by many different viral, bacterial, and fungal pathogens. Although several genera of fungi are capable of causing infections in the central nervous system (CNS), the most significant number of registered cases have, as causal agents, yeasts of the genus *Cryptococcus*. The relevance of cryptococcal meningitis has changed in the last decades, mainly due to the increase in the number of people living with human immunodeficiency virus/acquired immunodeficiency syndrome (HIV/AIDS) and medications that impair the immune responses. In this context, coronavirus disease 19 (COVID-19) has also emerged as a risk factor for invasive fungal infections (IFI), including fungal meningitis (FM), due to severe COVID-19 disease is associated with increased pro-inflammatory cytokines, interleukin (IL)-1, IL-6, and tumour necrosis factor-alpha, reduced CD4-interferon-gamma expression, CD4 and CD8 T cells. The gold standard technique for fungal identification is isolating fungi in the culture of the biological material, including cerebrospinal fluid (CSF). However, this methodology has as its main disadvantage the slow or null growth of some fungal species in culture, which makes it difficult to finalise the diagnosis. In conclusions, this article, in the first place, point that it is necessary to accurately identify the etiological agent in order to assist in the choice of the therapeutic regimen for the patients, including the implementation of actions that promote the reduction of the incidence, lethality, and fungal morbidity, which includes what is healthy in the CNS.

Meningitis and its agents

Meningitis is a potentially life-threatening infection characterised by the inflammations of the leptomeningeal membranes. The estimated annual prevalence of 8.7 million cases globally, and the disease is caused by many different viral, bacterial, and fungal pathogens.^1^

Fungal meningitis is a relatively uncommon infection that usually occurs in immunosuppressed individuals.^2^ Despite being known around 100 thousand species of fungi, about 300 are considered pathogenic, and of these, only about 10-15% of these usually produce systemic/ central nervous system (CNS) mycosis.^3^ Sub-acute symptoms, such as headache, neck stiffness, and fever, commonly occur, but their frequency varies according to the type of fungi involved. Additionally, fungal meningitis is often under recognised as a cause of meningitis worldwide.^4^

Although several genera of fungi are capable of causing infections in the CNS, the most significant number of registered cases has, as causal agents, yeasts of the genus *Cryptococcus*. The relevance of cryptococcal meningitis has changed in the last decades, mainly due to the increase in the number of people living with human immunodeficiency virus/acquired immunodeficiency syndrome (HIV/AIDS) and medications that impair the immune response. Corticosteroids, cytotoxic treatments for malignancies and therapies to prevent organ transplantation rejection, cause essential impacts on the immunomodulation of the immune response to fungi since it considerably impairs the adequate response in these infections.^5^ However, it is essential to mention that *Cryptococcus neoformans* and *Cryptococcus gattii* infection can cause a life-threatening condition in patients with intact and compromised immune systems.^6^

Nevertheless, it is not exclusive to cryptococcal infections. As previously mentioned, immunosuppressive conditions are directly associated with fungal infections, despite their increased frequency in immunocompetent patients. The reason why immunosuppressed patients are susceptible to fungal invasion of the CNS is due to compromised CD4+ T cell responses, since these cells play a surveillance role at blood brain barrier (BBB).^7^ Several opportunistic fungi can cause CNS infections, invading host epithelial cells and during haematogenous infection they can invade endothelial cells. These pathogens invade non-phagocytic host cells by inducing their own uptake, by transcellular, paracellular and transmigration pathways.^8^ These mechanisms are briefly showed in the Figure.^9^ ^-^ ^14^

**Figure:**
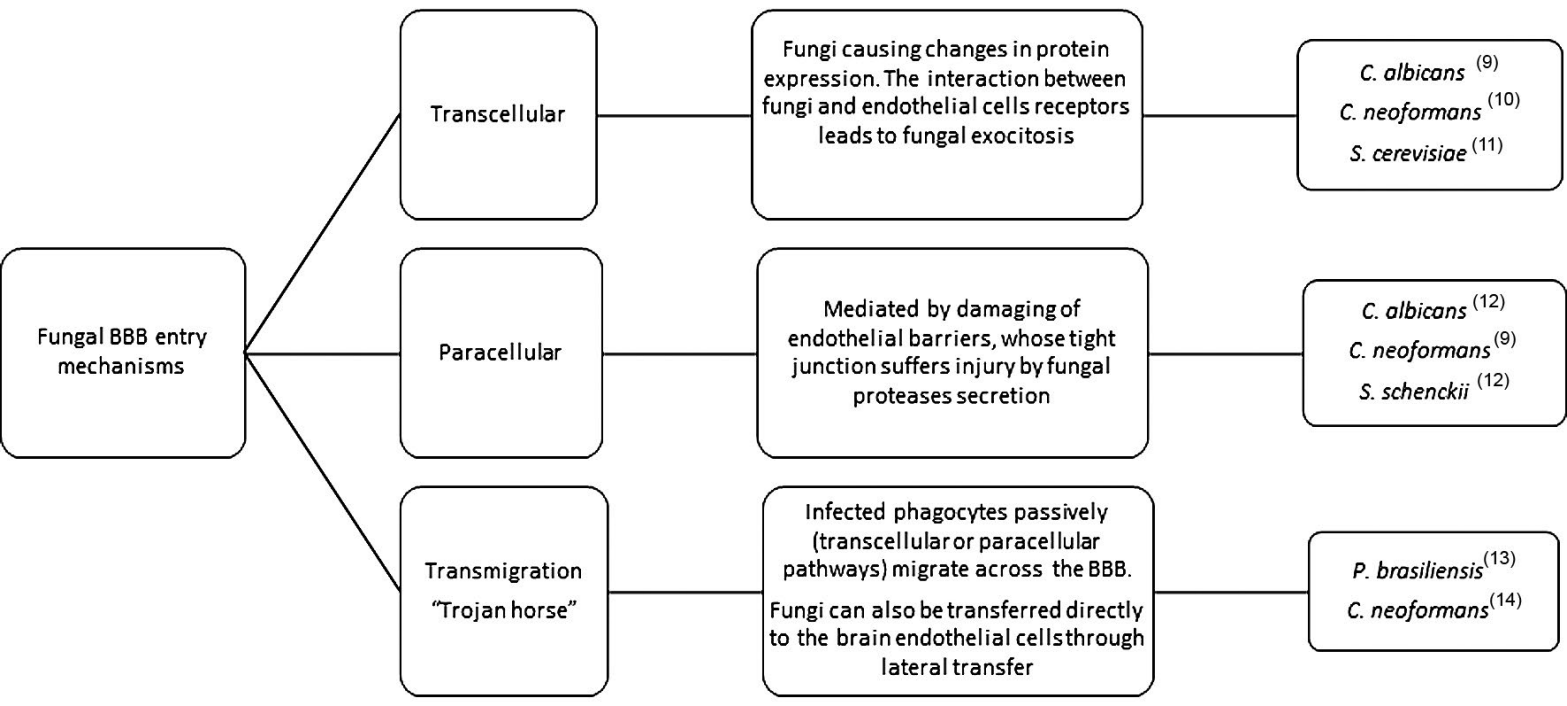
Schematic diagram of fungal blood brain barrier (BBB) entry mechanisms

Some mechanisms involved in the immune response to CNS fungal infection

Since the host immune response to fungi comprises resistance (the ability to limit fungal burden) and tolerance (the ability to restrict the host damage caused by the immune response or other mechanisms),^15^ and fungus are frequently exposed to humans, it is difficult to discriminate the healthy and pathogenic.^16^ In fungal infections, a predominantly Th1 response, characterised by the production of IL-2, IFN-γ and TNF-α, is related to protective immunity. On the other hand, the Th2-type response, characterised by the production of IL-4, IL-6 and IL-13, can promote deleterious effects, such as allergic responses or chronic infection.^17^

Despite of the impact of HIV/AIDS, malignancies or chronic poorly controlled medical conditions, such as diabetes mellitus, there are other important conditions that increase the risk of fungal infections, as genetic diseases, use of indwelling catheters and medications, especially to treat autoimmune disease, cancer or prevent transplant rejection.^18^

Different disorders of the immune system can increase the predisposition to infections by certain fungi. For example, primary immunodeficiency syndromes can favour infections by molds, especially in children. Uncontrolled diabetes is frequently associated to rhinocerebral infections. Disorders in the phagocytic function, as neutropenia, predispose patients to the development of CNS aspergillosis, as well as impairment of cell-mediated immunity predisposes patients to CNS cryptococcal, histoplasmal, coccidioidal, and blastomycotic infection.^18^ CNS infection by *Candida* sp, *Aspergillus* sp, and Zygomycetes is also frequently associated with impairment of granulocyte function.^18^

It is important to highlight that the increasing use of immunosuppressant and biologic agents as anti-tumour necrosis factor (TNF-α) and its therapies (methotrexate and infliximab) to treat neurologic diseases, such as neurosarcoidosis, has been associated with CNS histoplasmosis, coccidiomycosis, candidiasis, cryptococcosis, and aspergillosis, since this cytokine plays a crucial role in the formation/maintenance of granulomas as well as macrophage activation.^19^

Immunosenescence also contributes to the risk of fungal infection, due the reduction in naïve CD4^+^ and CD8^+^ T cells as well as decreased CD28 expression, which lead to a difficulty to recognise intracellular pathogens.^19^ Moreover, the consequence of the loss of functional T-cells is the increase of type 2 cytokines production, leading to a Th2 response and decreased Th1 response.^15^ ^,^ ^19^

The main host risk factors that can predispose to CNS fungal infections are summarised in Table I.

**TABLE I:** Host risk factors that predispose central nervous system (CNS) fungal infections

Predisposing factor	Fungal infections
AIDS	*C. neoformans*, *Candida* spp., *C. immitis*, *H. capsulatum*, *Aspergillus* spp., *B. dermatitidis*, *S. schenckii*
Infancy/Old age	*Candida* spp., *H. capsulatum*
Pregnancy	*C. immitis*
Diabetes mellitus	*Zygomycetes* spp., *Candida*
Intra-arterial or venous lines	*Candida* spp.
Intracranial shunt	*Candida* spp.
T-cell mononuclear phagocytic disorder	*C. neoformans*, *H. capsulatum*, *C. immitis*, *S. schenckii*
Cytotoxic chemotherapy/corticosteroids	*Aspergillus* spp., *Candida* spp., *Zygomycetes* spp.
Neutropenia	*Candida* spp., *Aspergillus* spp., *Zygomycetes*
Organ transplantation	*Aspergillus* spp., *Candida* spp., *C. neoformans*, *C. immitis*, *H. capsulatum*, *Zygomycetes* spp.

In the immunopathogenesis of CNS fungal infections are involved several mechanisms that coordinate the effectiveness of the host response. Although CNS has traditionally been regarded as an immunologically privileged site, when subjected to an injury or infection, it can mobilise and develop an immune response involving infiltrating CD4^+^ and CD8^+^ T cells, B-cells, macrophages, neutrophils and activated brain resident cells.^20^

Major histocompatibility complex (MHC) Class I and Class II molecules are expressed by CNS cells when a fungus activates resident cells. They can act as antigen-presenting cells (APC) and produce cytokines, chemokines and other molecules with antifungal activity.^20^ ^,^ ^21^ Nevertheless, T cell proliferation and cytokine secretion are stimulated by microglia, acting as antigen-presenting cells (APCs), in turn phagocytes are stimulated by T cells to ingest and eliminate fungal pathogens.^21^

The activation of microglia, and, consequently, the expression of immunostimulant and immunosuppressive cytokines in glial and immune cells, are factors that amplify or suppress the immune response in the CNS.^20^ ^,^ ^22^ Studies report the increase of IL-1α, IL-1β, IL-6, TNF-α, IFN-γ, IL-4 and IL-10 in patients with AIDS and meningeal cryptococcosis.^20^ ^,^ ^23^ IL-12, produced by monocytes, B-cells and activated microglial cells in the CNS, induces the production of IFN- γ by T-cells and natural killer (NK) cells and the development of a Th1 cellular immune response against infection, resulting in a positive effect on host immune response.^23^

Regarding TNF- α, like IL-1 is a major immune response-modifying cytokine produced primarily by activated macrophages but also by TCD4^+^ cells, and its early production is required to prevent the establishment of the fungus in the CNS.^23^ Finally, the role of IFN-γ has been shown to be important against *C. neoformans* and other fungal infections in CNS.^23^ This cytokine mediates protection due to the activation of effector cells already present at the site of infection or recruited to the site, because IFN- γ activates macrophages to better phagocyte fungi.^24^

Toll like receptors (TLRs) are pattern recognition receptors (PRR) that mediate cellular responses to pathogen-associated molecular patterns (PAMPs) in CNS fungal infections, binding to yeasts, conidia or hyphae by different pathways. Nevertheless, the less virulent strain of the fungus was preferably recognised by TLR2 and dectin-1, with balanced production of TNF-α and IL-10, while the more virulent strain induced production of only TNF-α. For example, intracellular *Cryptococcus* spp. is recognised by Toll-like receptor (TLR)-9 in APCs, which prime T-CD4^+^ lymphocytes, differentiate toward a Th1 phenotype.^25^ Additionally, TCD8^+^ lymphocytes are able to eliminate fungi by secretion of granulysin, a membrane-damaging molecule.^25^

Unlike observed in recognition of *Cryptococcus* spp., in *Paracoccidioides* spp. infection, TLR2, TLR4, and dectin-1 are also involved in the recognition and internalisation of the fungus. *Paracoccidioides* spp., are the most important genera, after *Cryptococcus* spp., involved in fungal CNS in Brazil, deploys similar strategies to avoid killing, as observed in other dimorphic fungi such as *Histoplasma* spp. and *Sporothrix* spp.^25^ ^,^ ^26^ A Th1 immune response pattern results in the formation of compact granulomas and control of fungal replication. On the other hand, developing Th2 and Th9 immune response patterns activates B lymphocytes, high levels of IgE subclass, hypergammaglobulinemia, and eosinophilia, leading to severe disseminated chronic form (CF). However, when the loss of Th1 function is compensated by the development of Th-17 and Th-22 responses, intense mucosal inflammatory responses rich in neutrophils can be triggered ^27^. Additionally, an essential subpopulation of T cells involved in the immunoregulation in CNS paracoccidioidomycosis is TCD4^+^CD25^+^FoxP3^+^ regulatory T cells (Treg), responsible for controlling the immune response and avoiding exacerbation of the inflammation and, consequently, the development of autoimmune diseases.^28^

As previously mentioned, the most significant number of registered cases of fungal meningitis has, as a causal agent, yeasts of the genus *Cryptococcus*. So, explaining some differences in pathogenesis among several fungal genera is essential. In HIV individuals, cerebral cryptococcosis is the most common opportunistic invasive infection. During recognition of the yeasts by the APCs, virulence factors as the presence of Glucuronoxylomannan and glucuronoxylomannogalactan in the capsule interferes with antigen presentation and skews the T cell response toward a nonprotective Th2 phenotype.^25^ ^,^ ^26^ In addition, laccase, an enzyme required for the melanin biosynthesis are known major virulence factors of *C. neoformans*, since its activity is capable to dampen Th17 responses and neutrophil accumulation and function during the early stages of an infection.^29^ Melanin in the cell wall of the fungus is also a virulence factor that plays an immunomodulation role, since it can reduce phagocytosis and oxidative burst.^16^ ^,^ ^25^ Additionally, melanisation of *C. neoformans* seems to be correlated with higher levels of IL-4 and MCP-1 and enhanced leukocyte recruitment.^29^

Highlighting the immune response to other important species, as an example, *Candida albicans*, the mechanism by which the fungus crosses the blood-brain barrier (BBB) and invades the CNS remains poorly understood. There are evidences that fungal endocytosis occurs from the interaction between Agglutinin-like protein 3 (Als3), a *C. albicans* invasion, and endothelial cells expressing Gp96.^30^ Microglia plays a central role in anti-*Candida* CNS host response, but also neutrophil recruitment is required to avoid uncontrolled CNS invasion. These cells are recruited mainly by Caspase Recruitment Domain 9 (CARD9), which encodes an adaptor downstream signal of fungal-sensing C-type lectin receptors, that releases interleukin (IL)-1b and IL-18 are released to activate inflammasome critical for protection against disseminated *C. albicans* infection.^30^ ^,^ ^31^

CARD9 is also critical in building an effective anti-*Aspergillus* CNS response, since CARD9-deficient patients were associated with impaired neutrophils accumulation.^32^ ^,^ ^33^ However, the immune response against *Aspergillus* infection starts with its recognition by (TLRs) and the lectin receptors, especially TLR2 and TLR4. It is interesting that *Aspergillus* morph type can modulate the inflammatory response since TLR2 recognise conidia and TLR4, hyphae.^17^ In this sense, *Aspergillus* conidia stimulate TLR2 and TLR4 receptors to induce an effective Th1 response, while hyphae lead to the loss of TRL4 and TLR2 signalling remains intact, stimulating the production of IL-10, an anti-inflammatory cytokine that directs the response to a non-protective Th2 profile. Thus, it is thought that *Aspergillus* invades through hyphal expansion that causes endothelial destruction by this mechanism of immune evasion.^15^ ^,^ ^18^ ^,^ ^33^

In this context, COVID-19 has also emerged as a risk factor to invasive fungal infections (IFI), including fungal meningitis (FM), due severe COVID-19 disease is associated with increased pro-inflammatory cytokines, interleukin (IL)-1, IL-6, and tumour necrosis factor alpha, reduced CD4- IFN-γ expression, CD4^+^ and CD8^+^ T cells.^34^ Additionally, the use of dexamethasone, largely used to treat COVID-19, interfere with lymph proliferative responses, probably by reducing the synthesis of IL-2, essential for combating IFI by clonal expansion of activated lymphocytes, especially TCD4^+^.^5^

Diagnosis

The diagnosis of FM represents a challenge since this CNS infection do not presents specific symptoms and signs of meningeal irritation and this difficulty in diagnosis is one of the factors that contribute to the increase in morbidity and mortality rates.^35^ As afore mentioned, cases of FM in immunocompetent patients have been more frequently reported in recent decades, especially those whose causal agents are *C. neoformans* and *Coccidioides immitis*.^35^

In the patients having or suspected of having fungal meningitis, the detection of fungi in cerebrospinal fluid (CSF) is scarce, with the exception of *C. neoformans*. Mainly for cryptococcal meningitis, the cryptococcal antigen (CrAg) lateral flow assay (LFA; Immuno-Mycologics, Norman, OK, USA) is the most rapid and effective way, presenting sensitivity and specificity being 99% in CSF.^3^

Currently, the gold standard technique for diagnosis is the identification by the isolation of the fungus in culture of the biological material, including CSF. However, the main disadvantage of this methodology is the required period of cultivation of the fungus, in many cases, is fairly accurate, results often take at least seven days, and so the clinical utility is limited since time is essential for the beginning of the treatment.^4^ ^,^ ^35^ Other techniques, as microscopy, histopathology and immunoassays still represent a strong reliance, while these techniques used on the front lines of diagnosing invasive fungal infection have not changed substantially in many years.^36^ Despite the importance of fungal pathogens as causes of meningitis, improved diagnostic tools are urgently needed in many cases due to the disease’s severity.^4^

For these reasons, in an ideal scenario, the fungal diagnosis comprises the interaction between foundation methodologies and high-technology molecular-based alternative technologies, as polymerase chain reaction (PCR), DNA-sequencing-based approaches and protein fingerprinting by matrix-assisted laser desorption ionisation time of flight (MALDI-TOF) mass spectrometry.^36^ However, the development of multiplex diagnostics that do not require fungal culture and include the simultaneous analysis of other important parameters, such as antifungal resistance, represents the future of diagnostic technologies.^36^

Despite being even rarer, several fungi have been either observed or isolated in cerebrospinal fluid specimens as *Acremonium* spp., *Aspergillus* spp., *Blastomyces dermatitidis*, *Candida* spp., *Coccidioides immitis*, *Histoplasma capsulatum*, *Paecilomyces variotii*, *Paracoccidioides brasiliensis*, *Schizophyilum* spp., *Sporothrix schenckii* and other *Cryptococcus* species.^37^ Nevertheless, since there are many types of fungal meningitis, diagnostic testing is not always effective, as observed in detecting *Blastomyces meningitis*, for example. So, some research groups propose applying different diagnosis methods.^4^ ^,^ ^38^ ^,^ ^39^

In a rapid systematic review of the literature from bibliographic databases (PubMed, Web of Science, LILACS, and Cochrane library), using the Medical Subject Headings (MeSH) terms “Fungal meningitis” 4,212 articles were found. After restricting the search strategy to Brazil and using additionally the “Central Nervous System Fungal Infections” MeSH term, this number decreases to 225 results [Supplementary data (Table)]. Based on these results, we evaluate the underreporting of fungal diseases and/or the need for an accurate diagnosis. In these articles, we selected studies that report non-*Cryptococcus* agents of CNS fungal infections, as shown in Table II.

**TABLE II:** Frequency of fungal agents reported by the included papers

Fungal agent	Articles published
*Paracoccidioides* spp.	14
*Sporothrix* spp.	8
*Histoplasma capsulatum*	6
*Aspergillus* spp.	2
*Aspergillus fumigatus*	2
*Trichosporon inki*	1
*Scedosporium apiospermum*	1
*Coccidioides* spp.	1
*Candida tropicalis*	1
*Candida parapsilosis*	1
*Blastomyces dermatitidis*	1
*Fonsecaea pedrosoi*	1
*Penicillium* spp.	1
*Conidiobolus coronatus*	1
*Rhodotorula glutinis*	1

It is possible to observe that *Paracoccidioides* spp., *Sporothrix* spp. and *Histoplasma capsulatum* are the main etiological agents of non-cryptococcal CNS in Brazil, respectively. In this sense, it is important to discuss the diagnosis methods with higher sensibility, specificity, applicability, low cost, accuracy, short time, with the aim to improve the management of the disease.

The serological diagnosis of paracoccidioidomycosis (PCM) is an example of the importance of an accurate identification of the etiological agent to better manage the disease and how the same peptide can be used both as a diagnostic target and as a therapeutic alternative. The glycoprotein Gp43 produced by PB (PBGp43) is one of the main serological markers used in the diagnosis of PCM. However, *Paracoccidioides lutzii* (PL) expresses low amounts of Gp43 and PLGp43 displays few epitopes in common with the immunodominant PBGp43, compared to *P. brasiliensis*, which impair the efficiency of serological diagnosis in patients infected with PL. In addition, the peptide 10 (P10) from the PBGp43 has been explored as a therapeutic, since it can induce protective immune responses in *in vitro* and *in vivo* models due its ability to increase proliferation of cells T CD4^+^ Th1 and the expression of high levels of INF-γ and IL-2.^40^

From the perspective of the immunoassays, the use of a chromogenic quantitative enzyme designed to detect 1,3-beta-D-glucan (a component of the fungal cell wall) BDG (in ng/ml), is one promising test.^4^ Several studies have been conducted to evaluate the efficacy of this test on serum samples to diagnose invasive fungal infections,^41^ ^,^ ^42^ and more recently, studies have investigated the use of BDG testing on CSF^4^ to identify *Candida* spp., *Aspergillu*s spp. and *Exserohilum* spp. as agents of FM.

We can also cite the use of Nested PCR to identify *Sporothrix sensu lato* in CSF. The authors demonstrate that, in cases of low fungal burden that leads to a negative culture, molecular tools are essential to detect and identify the causal agent of the disease.^38^ Also, they reinforce that this approach for a known technique is innovative and has the benefit of improving diagnosis and early treatment in patients with meningoencephalitis due to *Sporothrix sensu lato*.^38^

The same group has been demonstrated another tool that can apply for diagnosis of bloodstream infection with brain involvement: polyphasic taxonomy. In the study published by Oliveira and collaborators,^39^ *Schizophyllum commune* in an HIV-infected patient was identified as etiological agent of bloodstream infection with brain abscess. The fungus was subcultured in potato dextrose agar (PDA), and after a week, only in blood sample, was seen, macroscopically, a cottony white colony that turned light grey and hyaline, septate, with no dichotomously branching hyphae in microscopy. Since it was not possible to achieve fungal identification only with conventional mycological techniques, partial sequencing of the internal transcribed spacer (ITS) region of ribosomal DNA (rDNA) was performed using ITS1 (TCCGTAGGTG AACCTGCGG) and ITS4 (TCCTCCGCTTATTGATATGC).^39^

The newest molecular test to diagnose infections in CNS is named FilmArray (USA), was approved by FDA in 2015, and can detect 14 pathogens commonly involved in CNS infections, such as *C. neoformans* and *C. gattii*. Nonetheless, in an elegant systematic review, Tansarli and Chapin^43^ concluded that the highest proportions of false negative is related to FM mostly patients with antigen detected, either treatment or cleared disease.

In conclusions, this article highlights, in the first place, which a precise diagnosis is required to assist in choosing the best therapeutic regimen for the patient, if possible, with the species. In this sense, accurate molecular diagnostic tests and fast have a key role in patients with fungi meningitis. Additionally, knowing the mechanisms of the immune response to fungal infections is essential not only to identify risk factors for certain infections but also to increase treatment options for more severely ill patients, for example, alternatively or concomitantly to conventional antifungal drug treatments.

Finally, due to the impact of COVID-19 in the world and climate changes, we reinforce the importance of paying attention to changes in the virulence profile of some fungi which, has emerged with pathogenic species, increasing the incidence, lethality, and morbidity of fungal diseases, especially those that affect the CNS.
