# Supplementary material for: Cerebrospinal fluid: a target of some fungi and an overview
Source: Mem Inst Oswaldo Cruz. 2023 Mar 20;118:e220251. doi: 10.1590/0074-02760220251 (PMC10027065; doi:10.1590/0074-02760220251)
Supplement: Supplementary file 1 [file 1678-8060-mioc-118-e220251-s.pdf]

TABLE

PRISMA 2020 flow diagram for new systematic reviews which included searches of databases, registers and other sources

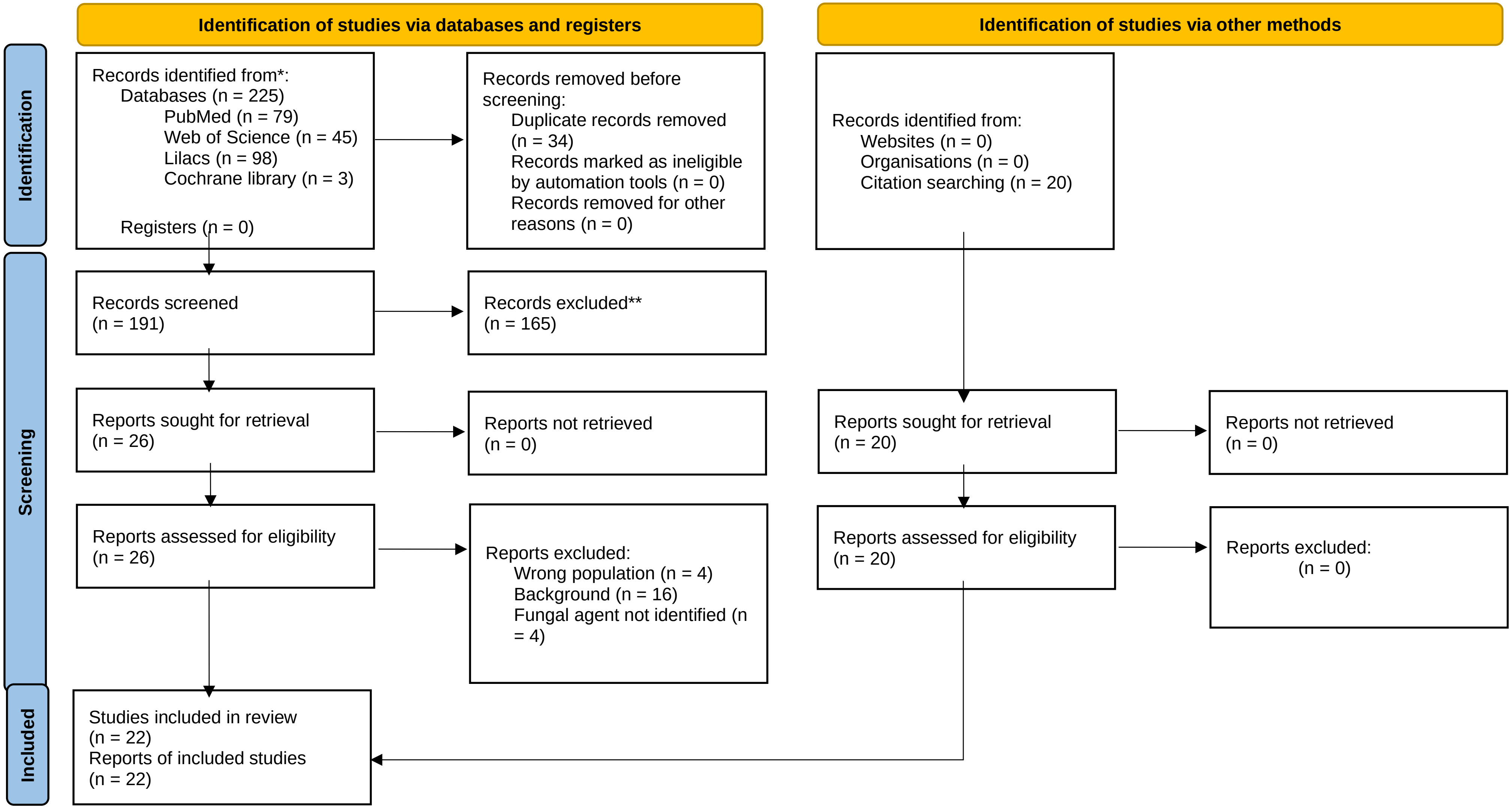

*Consider, if feasible to do so, reporting the number of records identified from each database or register searched (rather than the total number across all databases/registers); **If automation tools were used, indicate how many records were excluded by a human and how many were excluded by automation tools.
Source: Page MJ, McKenzie JE, Bossuyt PM, Boutron I, Hoffmann TC, Mulrow CD, et al. The PRISMA 2020 statement: an updated guideline for reporting systematic reviews. BMJ. 2021; 372: n71. doi: 10.1136/bmj.n71. For more information, visit: http://www.prisma-statement.org/.

CC BY
